# A scoping review of prevalence, incidence and risk factors for HIV infection amongst young people in Brazil

**DOI:** 10.1186/s12879-017-2795-9

**Published:** 2017-10-11

**Authors:** Igor Pedrosa Saffier, Hélia Kawa, Guy Harling

**Affiliations:** 1 Department of Epidemiology, Fluminense Federal University, Niterói, Brazil; 2 Institute for Global Health, University College London, Mortimer Market Centre, London, WC1E 6JB UK; 3 Department of Global Health and Population, Harvard T.H. Chan School of Public Health, Boston, USA

**Keywords:** Brazil, HIV, Adolescents, Young adults, Review

## Abstract

**Background:**

Despite young people being a key population for HIV prevention, the HIV epidemic amongst young Brazilians is perceived to be growing. We therefore reviewed all published literature on HIV prevalence and risk factors for HIV infection amongst 10-25 year olds in Brazil.

**Methods:**

We searched Embase, LILACS, Proquest, PsycINFO, PubMed, Scopus and Web of Science for studies published up to March 2017 and analyzed reference lists of relevant studies. We included published studies from any time in the HIV epidemic which provided estimates specific to ages 10-25 (or some subset of this age range) for Brazilians on either: (a) HIV prevalence or incidence; or (b) the association between HIV and socio-demographic or behavioral risk factors.

**Results:**

Forty eight publications met the inclusion criteria: 44 cross-sectional, two case-control, two cohort. Four studies analysed national data. Forty seven studies provided HIV prevalence estimates, largely for six population subgroups: Counselling and Testing Center attendees; blood donors; pregnant women; institutional individuals; men-who-have-sex-with-men (MSM) and female sex workers (FSW); four provided HIV incidence estimates. Twelve studies showed HIV status to be associated with a wide range of risk factors, including age, sexual and reproductive history, infection history, substance use, geography, marital status, mental health and socioeconomic status.

**Conclusions:**

Few published studies have examined HIV amongst young people in Brazil, and those published have been largely cross-sectional and focused on traditional risk groups and the south of the country. Despite these limitations, the literature shows raised HIV prevalence amongst MSM and FSW, as well as amongst those using drugs. Time trends are harder to identify, although rates appear to be falling for pregnant women, possibly reversing an earlier de-masculinization of the epidemic. Improved surveillance of HIV incidence, prevalence and risk factors is a key component of efforts to eliminate HIV in Brazil.

## Background

Young people, especially young women, are considered a key population for HIV prevention interventions worldwide [1]. However, targeting interventions is difficult when information on HIV prevalence and risk factors is patchy or missing altogether. Several risk factors for sexually transmitted infections (STIs) are elevated amongst young people, such as being in the beginning of their sexual life, experimenting with high-risk behaviors and feeling invulnerable [2]. Although Brazil is world-renowned for its leadership in the fight against AIDS [3–5], and even as AIDS rates are declining in many other places, Brazil is perceived to be facing a sharp increase in HIV infections among young people [6].

AIDS has been a reportable condition in Brazil throughout the epidemic. Over the past 10 years the AIDS detection rate has averaged 20.5 cases per 100,000 persons per year [7]. Between 2004 and 2013, reported AIDS cases in Brazil rose by 53.2% among those aged 15-19 and 10.3% among those aged 20-24 [8]. These increases were greater in men than in women: between 2005 and 2014 reported AIDS case rates per 100,000 persons per year rose from 2.1 to 6.7 for 15-19 year old males and from 3.4 to 4.2 among 15-19 year old females; for 20-24 year olds the rate rose from 16.0 to 30.3 among men but decreased from 15.3 to 12.0 among women [7]. These differences in case rate trends are reflected in the changing ratio of male to female AIDS notifications: among 13-19 year olds this ratio fell from 2.7:1 in 1990 to a low of 0.6:1 in 2005, before rebounding to reach 1.6:1 in 2014. Notably, these rising rates are in contrast to older ages: AIDS notification rates fell in all five-year age ranges from 30 to 49 years old between 2005 and 2014.

In contrast, HIV has not historically been a reportable condition. From 2007 to June 2015, 93,260 HIV infections were notified in Brazil [7], however, mandatory notification of HIV infection began only in June 2014. Additionally, health service providers can notify both newly-identified and existing known cases, which makes epidemiologic analysis of national HIV case reports difficult. In 2015, approximately 830,000 people were estimated to be living with HIV in Brazil, a prevalence of 0.40% [8]. Between 2007 and 2015, the proportion of HIV-positive individuals reported to be the age groups 10-14, 15-19 and 20-24 years old rose from 0.3%, 4.3% and 13.4% of all notifications to 0.3%, 6.1% and 18.2%, respectively. Across all ages, the male to female ratio of notified HIV infections increased slightly from 1.9 in 2007 to 2.2 in 2014.

Given these epidemiological patterns, a review of the literature on HIV in young people in Brazil seems timely. Past literature reviews have discussed specific aspects of HIV in adolescents and young people in Brazil. These studies have shown the difficulty of transitioning from childhood to adult life for adolescents living with AIDS [9], the efficacy of preventive interventions focusing on this population [10], the situation of orphans and vulnerable children [11] and the relationship between STIs, AIDS and abuse of psychoactive substances in adolescence [12]. In addition, several articles have analyzed risk behavior and HIV infection among adolescents in Brazil in specific populations, such as users of anonymous Counselling and Testing Centers (CTA), interns of the correctional system, pregnant women, men-who-have-sex-with-men (MSM) and others. However, there is no comprehensive literature review putting together the results of these studies amongst young people, and it is difficult to see trends over time in the adolescent HIV epidemic in Brazil.

The aim of this study is therefore to review all published evidence on HIV prevalence and incidence, and how they relate to risk behaviors among different populations of Brazilian adolescents between 1982 and 2015. Such information should help identify gaps in the literature, and provide a scientific basis for the development of preventive strategies for this age group.

## Methods

We performed a systematic search on seven electronic databases – PubMed, Embase, PsycINFO, LILACS, Web of Science, Scopus and ProQuest – to identify potentially relevant analyses. The keywords we used for this search were the MeSH terms [“HIV” or “HIV infection”], “Adolescents” and “Brazil” or similar non-MeSH terms outside of PubMed. The search was conducted between March 25th and March 31st 2017. Reference lists were also analyzed for any potentially relevant articles not included in the original search. We included any conference proceedings (within Web of Science and Scopus) and dissertations (ProQuest) found in our database searches.

One author analyzed all articles found by title to select those that were potentially relevant, with a strong bias towards retention. The abstracts of all studies selected based on their titles were independently evaluated by two authors (IPS and GH) and any discrepancies were kept in the analysis. For all studies selected at the abstract stage, data were extracted using an instrument designed for this study, covering sociodemographic characteristics (gender, age group, location, race and social categories), methodology (study design, study population, data source, time period of data collection, baseline sample size and loss to follow up), and outcomes (HIV prevalence or HIV incidence, risk behaviors).

The final decision to include studies was made based on this data extraction and whether it met the inclusion/exclusion criteria, based on independent evaluation by two authors (IPH and GH), and a discussion of any discrepancies; the third author (HK) was available for consultation if agreement could not be reached. Our inclusion criteria were that studies: (i) contained either (a) HIV prevalence or incidence data or (b) analysis of risk factors for HIV infection; (ii) either focused on individuals aged between 10 and 25 years, or stratified their results by the age group of interest; (iii) included data on the Brazilian population. Conversely, our exclusion criteria were: (i) lack of stratification by age, if covering a broader age group than 10-25 years; (ii) lack of stratification by country, if a multinational study; (iii) lack of quantitative presentation of data on prevalence, incidence or risk factors; (iv) reporting only AIDS cases instead of HIV infections; (v) reporting only on HIV positive individuals.

### Analysis

First, we evaluated HIV prevalence in different population groups. The analysis started with broader groups, closer to the national population level, such as users of CTAs, blood donors and pregnant women. We then progressed to more specific groups such as residents at correctional institutions, sex workers, MSM and people who inject drugs (PWID). Second, we analyzed the association between various exposures and HIV prevalence. These exposures included sociodemographic characteristics – such as age, place of living and marital status – and behavioral characteristics such as age of sexual debut, drug use, sexual preferences and sex in exchange for money.

We presented all crude HIV prevalence and incidence rates reported in the studies, so long as they provided a number specific to the age range of interest. For risk factors, we presented any exposure reported to be significantly associated with HIV infection in the relevant age range. We preferentially reported adjusted measures of association and confidence intervals when provided. Finally, when no risk factors were significantly associated, we noted this.

We did not appraise the methodological quality or risk of bias of the included articles, which is consistent with guidance on scoping review conduct [13]. All authors contributed to the elaboration of the discussion of the article through bibliographic search and their expertise.

## Results

**Fig. 1:**
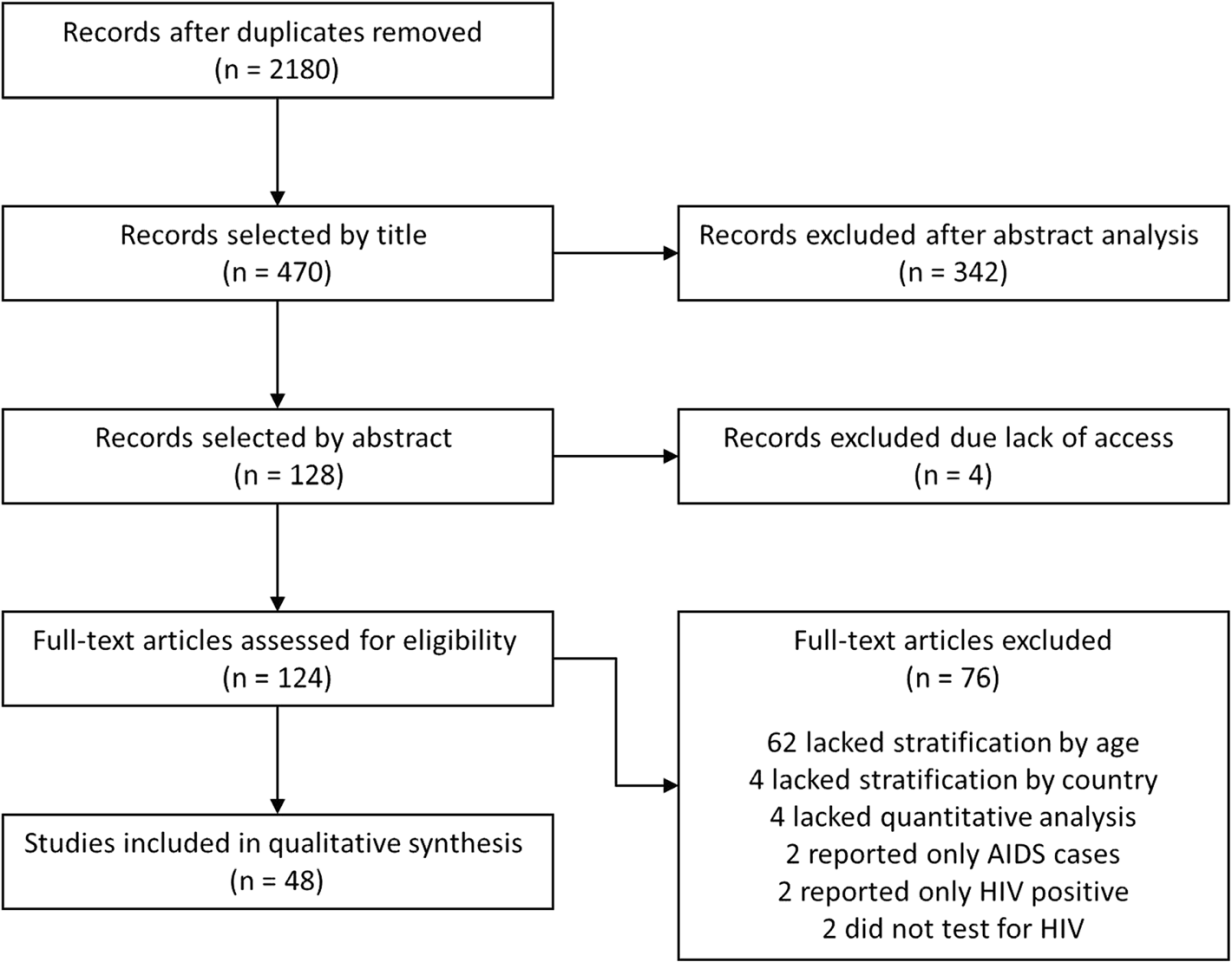
Flow diagram of systematic review process

Of the 2180 unique articles identified by database searches, 470 studies were selected as potentially relevant for this analysis based on their titles (Fig. 1). We retained 128 of these based on their abstracts, although we were unable to obtain the full text of four of these (all four were published before 1995). Sixty two studies that otherwise met our inclusion criteria were excluded because they did not stratify their results by age group so we could extract data specific to young people, and 14 other articles did not meet other inclusion criteria. This left 48 studies which reported either HIV prevalence or risk factors for our age group of interest and were published in English, Portuguese or French (note, we kept one study with an age range 15-26). Thirty-six of these 48 articles provided an age-specific HIV prevalence, but not HIV risk factors; we therefore report results separately for prevalence and risk exposures. We summarize all studies included in the final analyses in Table 1.

**Table 1:** Summary of all studies included in the systematic review

First Author	Location	Sample (N)	Data collection	Design	Main Results	Reference
Pechansky	Porto Alegre RS	CTA attendees (1026; 390 aged <25)	1995; 1997	Repeated cross-section	HIV prevalence for <25 year olds: 11.5%	[14]
Alves	Santos, SP	CTA attendees (7794; 2769 aged <25)	1996-1999	Cross-section	HIV prevalence for <25 year olds: 3.1% Recent HIV infection for <25 year olds: 0.3% (based on STARHS algorithm)	[15]
Bassols	Porto Alegre RS	Adolescent CTA attendees (287)	2000-2001	Cross-section	HIV prevalence among boys: 4.8% HIV prevalence among girls: 7.4%	[16]
de Araújo	Campos dos Goytacazes RJ	CTA attendees (7386; 1129 aged 13-19; 1878 aged 20-24)	2001-2002	Cross-section	HIV prevalence for 13-19 year olds: non-pregnant women: 0%; pregnant women: 0.5%; men: 12.1% HIV prevalence for 20-24 year olds: non-pregnant women: 5.8%; pregnant women: 0.47%; men: 6.4%	[17]
Bassols	Porto Alegre RS	Female adolescent CTA attendees (258)	2000-2001	Cross-section	HIV prevalence: 7.4% Risk factors: Composite drug risk: using illicit drugs & visiting drug using/selling places (aOR: 4.18, 95%CI: 1.47-11.8)	[18]
Bassols	Porto Alegre RS	Adolescent CTA attendees (402)	Not reported	Cross-section	HIV prevalence: 6.2% Risk factors: SCL-90-R psychiatric score (aOR: 1.88, 95%CI: 1.06-3.34); Composite sexual behavior risk (aOR: 1.63, 95%CI: 0.98-2.70)	[19]
Cook	Rio de Janeiro RJ	Women attending CTA (200; 44 aged 14-19; 97 aged 20-24)	2001	Cross-section	HIV prevalence for 14-19 year olds: 6.8% HIV prevalence for 20-24 year olds: 9.3%	[20]
Bassichetto	Sao Paulo SP	Attendees of 4 CTAs who tested positive for HIV-1 (485; 14 aged 14-19.9; 82 aged 20-24.9)	2002-2004	Cross-section	Risk factors: Recent infection not associated with age: (14-19 years old: 28.6%; 20-24 years old: 24.4%)	[21]
de Souza	Goias state and Federal District	Attendees of 15 CTAs (16,991; 784 male and 1652 females aged 13-19)	2003-2004	Cross-section	HIV prevalence for males aged 13-19: 1.1% HIV prevalence for females aged 13-19: 0.2%	[22]
Monteiro	Feira de Santana, BA	Attendees of the municipal CTA aged 11-18 (3482; 664 male, 1183 non-pregnant female, 1635 pregnant female)	2003-2012	Cross-section	HIV prevalence for males: 1.08% HIV prevalence for non-pregnant females: 1.05% HIV prevalence for pregnant women: 0.31%	[23]
de Castro	Rio de Janeiro, RJ	Attendees of 3 CTAs (9008; 2525 aged <25)	2004-2005	Cross-section	HIV prevalence for <25 year olds: 2.6% HIV incidence for <25 year olds: 0.6%/year (based on BED-CEIA test)	[24]
Scheineder	Santa Catarina state	Attendees of 14 CTAs (22,846; 2416 aged 10-19)	2005	Cross-section	HIV prevalence for females aged 10-19: 0.9% HIV prevalence for males aged 10-19: 2.1%	[25]
Cavalcanti	Recife PE	Attendees of 5 CTAs (32,256; 16,161 aged <25)	2007-2009	Cross-section	HIV prevalence for <25 year olds: 0.82% (95%CI: 0.68-0.97%) Recent infection for <25 year olds: 12/27 prevalent cases (based on BED-CEIA test)	[26]
Pereira	Feira de Santana BA	Attendees of the municipal CTA (3768)	2007-2011	Cross-section	HIV prevalence for males: 3.0% HIV prevalence for females: 1.6% Risk factors for females: drug use (PrR = 2.1, 95%CI: 1.15-3.82); alcohol use (PrR = 2.1, 95%CI: 1.16-3.91); married (PrR = 2.02, 95%CI: 1.09-3.75) Risk factors for males: use of drugs other than alcohol (PrR: 13.25, 95%CI: 5.12-34.28); MSM (PrR: 5.21, 95%CI: 2.57-10.57)	[27]
Andrade Neto	Curitiba PR	Blood Donors (213,666; 177 aged <18, 51,670 aged 18-25)	1992-1999	Cross-section	HIV prevalence for <18 year olds: 0.56% HIV prevalence for 18-25 year olds: 0.14%	[28]
Kupek	Santa Catarina state	Blood Donors (293,725; 95,797 aged 16-24)	2007-2013	Cross-section; Cohort	HIV prevalence for 16-24 year olds: 1.22% (95%CI: 1.01-1.46) HIV incidence for 16-24 year olds: 0.28 per 100PY (95%CI: 0.20-0.37) Seroprevalence rose throughout the study period for males but for females it rose only until 2012, then declined.	[29]
Souza	Recife PE	Pregnant women seeking antenatal care (1000; 0.9% aged <15, 31.6% aged 15-20)	1993	Cross-Section	0 pregnant women aged <21 out of ~325 were HIV+	[30]
de Freitas Oliveira	Sao Paulo SP	HIV-positive pregnant women seeking antenatal care (106; 10 aged 15-19, 28 aged 20-24)	1991-2002	Cross-section	Recent HIV infection for 15-19 year olds: 10% Recent HIV infection for 20-24 year olds: 17.9% (based on STARHS algorithm) No significant association between age and recent infection status	[31]
Reiche	Londrina PR	Pregnant women at a teaching hospital (1473; 290 aged 10-20)	1996-1998	Cross-section	HIV prevalence for 10-20 year olds: 1.0%	[32]
Souza	Campina Grande PB	Pregnant women at prenatal services (386; 127 aged 14-19; 140 aged 20-25)	2001	Cross-section	HIV prevalence for 14-19 year olds: 0.0% HIV prevalence for 20-25 year olds: 0.07%	[33]
de Macedo Orione	Cuiabá MT	Postpartum women (1607; 575 aged 15-20; 525 aged 21-25)	2001-2002	Cross-section	HIV prevalence for 15-20 year olds: 0.5% HIV prevalence for 21-25 year olds: 0.4%	[34]
Figueiró-Filho	Campo Grande, MS	Pregnant women at prenatal services (35,512; 9906 aged 11-20)	2002-2003	Cross-section	HIV prevalence for <21 years old: 0.2% No significant association between age and HIV serostatus	[35]
Cardoso	27 Southern Brazil cities	Pregnant women attending CTAs (8002; 4630 aged 12-25)	2003	Cross-section	HIV prevalence for 12-25 year olds: 0.5%	[36]
Costa	Goiania GO	Pregnant women at prenatal services (28,561, 6664 aged 12-19)	2004-2005	Cross-section	HIV prevalence for 12-19 year olds: 0.03%	[37]
Costa	Feira de Santana BA	Pregnant women aged <25 at prenatal services (3030)	2004 - 2008	Cross-section	HIV prevalence for ≤16 year olds: 0.3% HIV prevalence for 17-19 year olds: 0.5% HIV prevalence for 20-24 year olds: 0.1%	[38]
Pinho-Pompeu	Campinas, SP	Pregnant women at prenatal services (458 aged 10-19)	2005-2013	Cross-section	HIV prevalence for 10-19 year olds: 1.97% There was a positive association between anemia and HIV infection (*p* = 0.02)	[39]
de Melo Inagaki	Sergipe state	Pregnant women at prenatal services (9550; 24.9% aged 10-19)	2007	Cross-section	HIV prevalence for 10-19 year olds: 0.09%	[40]
Moura	Maceió, AL	Pregnant women at prenatal services (54,616; 17,231 aged <19)	2007-2012	Cross-section	HIV prevalence for <19 year olds: 0.3%	[41]
Miranda	National	Women in labor at public hospitals (2071)	2009	Cross-section	HIV prevalence: 0.7% Risk factors: Living in the North region (aOR: 2.0 95%CI: 1.07-3.73); STI history (aOR: 42.5, 95%CI: 1.89-168.49)	[42]
Ferezin	29 cities in Paraná state	Pregnant women at a teaching hospital (1534; 354 aged 14-19)	2010	Cross-section	HIV prevalence for 14-19 year olds: 0.3%	[43]
Domingues	National	Pregnant women (23,894; 4570 aged 12-19)	2011-2012	Cross-section	HIV prevalence for 12-19 year olds: 0.14%	[44]
Pinto	Belo Horizonte MG	Inmates in a youth correctional institute (394; 195 previously street-based, 199 previously home-based)	1989-1991	Case-control	HIV prevalence for street-based youth: 2% HIV prevalence for home-based youth: 0% Risk factors: 2 of 3 HIV-positive males reported using injection drugs; all HIV-positive youths reported heterosexual activity	[45]
Zanetta	Sao Paulo SP	Inmates in a youth correctional institute (1215)	1994	Cross-section	HIV prevalence for females: 10.3% HIV prevalence for males: 2.6% Risk factors for females: Commercial sex work (OR = 5.98 (95%CI: 1.04-34.30) Risk factors for males: HCV seropositivity [OR = 26.5 (95%CI: 8.83-79.70)]; age > 18 [OR = 3.45 (95%CI: 1.21-9.86)]; PWID [OR = 3.39 (95%CI: 1.10-10.4)	[46]
Coelho	Ribeirão Preto, SP	Inmates in a correctional institute (333; 96 aged <25)	2003	Cross-section	HIV prevalence for 19-24 year olds: 0.09%	[47]
Fialho	Salvador BA	Incarcerated youth aged 11-18 (297)	2004-2005	Cross-section	HIV prevalence: 0.34%	[48]
Harrison	Rio de Janeiro, RJ	High-risk HIV- MSM recruited at HIV testing sites and MSM venues (750; 242 aged <25)	1995-1997	Cohort	HIV incidence for <20 year olds: 8.4 (95%CI: 1.7-15) per 100PY HIV incidence for 20-24 year olds: 3.9 (95%CI: 1.7-6.1) per 100PY Age < 25 was associated with HIV seroconversion (aRR = 2.6, 95%CI: 1.3-5.6)	[49]
Szwarcwald	National	Military conscripts (1997: 9844; 1998: 30,318; 1999: 29,373; 2000: 23,659; 2002: 30,970)	1997-2002	Cross-section	HIV prevalence (2002): 0.09% Risk factors: positive syphilis test OR = 5.72 (95%CI: 1.32-24.9), MSM OR = 4.06 (95%CI: 1.29-12.8), At least 1 STI related problem OR = 2.76 (95%CI: 1.18-6.45), More than 10 lifetime sexual partners OR = 2.33 (95%CI: 1.05-5.18), Resident of Southern Brazil OR = 2.77 (95%CI: 1.10-6.99)	[50]
Soares	Campinas SP	MSM (658; 167 aged 14-19, 190 aged 20-24)	2005-2006	Cross-section	HIV prevalence for 14-19 year olds: 2.9% HIV prevalence for 20-24 year olds: 5.9%	[51]
Szwarcwald	National	Military conscript personnel aged 17-21 (35,432, of whom ~800 report being MSM)	2007	Cross-section	HIV prevalence overall: 0.1% HIV prevalence for MSM: 1.2% Risk factors: being MSM OR = 11.16 (95%CI: 4.90-25.39); having at least one STI-related problem OR = 2.53 (95%CI: 1.20-5.36); >10 lifetime partners OR = 2.52 (95%CI: 1.21-5.25)	[52]
Guimarães	Belo Horizonte, MG	MSM (272; 113 aged <24)	2010	Cross-section	HIV prevalence for <24 year olds: 2.8%	[53]
de Souza	São Paulo, SP	MSM (771; number aged <25 not reported)	2011-2012	Cross-section	HIV prevalence for 18-24 year olds: 6.4% (95%CI: 3.5-11.5%)	[54]
Trevisol	Imbituba SC	Female sex workers (90; 44 aged <26)	2003-2004	Cross-section	HIV prevalence for <26 year olds: 6.8%	[55]
Schuelter-Trevisol	Santa Catarina state	Sex workers (147; 57 aged 18-24)	2009	Cross-section	HIV prevalence for 18-24 year olds: 5.3%	[56]
Costa	Porto Alegre, RS	Male to Female transsexuals (284; 128 aged 15-26)	1998-2014	Cross-section	HIV prevalence for 18-26 year olds: 14.8%	[57]
Freitas-Carvalho	Rio Branco, AC	Attendees of immunization campaign (390; 118 aged 12-21)	1999	Cross-section	HIV prevalence: 0%	[58]
Codes	Salvador, BA	Women attending a public family planning clinic (202; 70 aged <22; 77 aged 22-25)	Not reported	Cross-section	HIV prevalence for <21 year olds: 0% HIV prevalence for 22-25 year olds: 4%	[59]
Szwarcwald	Recife, PE and Curitiba, PR	General population (902 in Recife; 1013 in Curitiba)	2013	Cross-section	HIV incidence for 13-24 year olds in Curitiba, PR: 0.060%/year (18.8% of all HIV-positive) HIV incidence for 13-24 year olds in Recife, PE: 0.059% (19.4% of all HIV-positive)	[60]
Silveira	Pelotas RS	HIV-positive women (144; 11 aged 15-19, 39 aged 20-24); AIDS-diagnosed women (130; 7 aged 15-19, 13 aged 20-24); door-to-door interviewed controls (1537; 151 aged 15-19, 240 aged 20-24)	1999-2000 (controls); 2003-2004 (cases)	Case-control	Risk factors: Odds of being HIV-positive were higher for 15-19 year olds (OR: 3.0, 95%CI: 1.4-6.6) and for 20-24 year olds (OR: 6.2, 95%CI: 1.4-11.4) than for those aged ≥40	[61]

All sample sizes cited are analytic, and thus do not include non-respondents. *CTA* Counselling and testing center, *MSM* Men who have sex with men, *PWID* People Who Inject Drugs, *STI* Sexually Transmitted Infection, *STARHS* Serologic Testing Algorithm for Recent HIV Seroconversion, *OR* Odds Ratio, *aOR* Adjusted Odds Ratio, *PrR* Prevalence Ratio, *100PY* 100 person-years

### Prevalence and incidence

Forty-seven studies provided age-specific HIV prevalence or incidence estimates.

#### Counselling and testing Center attendees

Fifteen studies studied counselling and testing center (CTA) attendees without focusing only on pregnant women. The HIV seroprevalence of individuals attending CTAs in Porto Alegre in 1995 and 1997 was 11.5% amongst under 25 year olds [14]. Young age was found to be protective for HIV infection (adjusted Odds Ratio [aOR] for 25-60 vs <25: 1.7; 95%CI: 1.1-2.7), however this study was not stratified by gender. The HIV prevalence among clients aged under 25 years old in a CTA in Santos, SP between 1996 and 1999 was 3.1%, of which 0.3% were diagnosed as recently infected based on the serologic testing algorithm for recent HIV seroconversion (STARHS) [15]. Adolescents aged 13-20 attending a CTA in Porto Alegre, RS in 2000-01 had an overall HIV seroprevalence of 6.4%: 4.8% among males; 7.2% among females [16]. Between 2001 and 2002, users of a CTA in Campos dos Goytacazes, RJ aged 13 to 19 had a seroprevalence of 0% among non-pregnant women, 0,50% among pregnant women and 12,1% among men [17]. In addition, amongst 20 to 24 years old, non-pregnant women had a prevalence of 5,8%, pregnant women 0,47% and men 6,4%. Female members of an expanded sample of 13-20 year old females attending this CTA over the same period had an HIV prevalence of 7.4% [18]. The same authors report an HIV prevalence of 6.2% in another overlapping group of adolescents visiting the clinic [19], although they did not report when the data was collected within the article. Women attending a CTA in Rio de Janeiro in 2001 had an HIV prevalence of 6.8% amongst 14-19 year olds and 9.3% amongst 20-24 year olds [20]. Between 2002 and 2004, Bassichetto et al. found that among HIV-positive adolescents and young adults, 28.6% of 14-19 year olds and 24.4% of 20-24 year olds were diagnosed as recently infected based on STARHS, and that these proportions were higher than in older age groups [21].

In 2003-04 HIV prevalence in CTAs in Goiás and Federal District states in Central Brazil was 1.1% among males and 0.2% among females aged 13-19 years [22]. From 2003 to 2012, HIV prevalence amongst 11-18 year olds attending CTAs in Feira de Santana, BA was 1.08% for males and 1.05% for non-pregnant women [23].Between 2004 and 2005, the prevalence of HIV infection among people aged under 25 years old testing in a CTA in Rio de Janeiro, RJ, was 2.6% (95%CI: 1.9-3.2) and the estimated incidence (based on IgG BED capture enzyme immunoassay [BED-CEIA]) was between 0.56 and 0.87%, depending on the estimation method used [24]. In 2005, females and males aged 10-19 years old attending CTAs in Santa Catarina had an HIV prevalence of 0.9% (95%CI: 0.5-2.3) and 2.1% (95%CI: 1.1-3.1), respectively [25]. Adolescents represented 16.3% of females and 9.5% of males who accessed these services, and their prevalence was lower than the overall prevalence of 2.0% among women and 5.6% among men. In 2007-2009, HIV prevalence among users of CTAs under 25 years in Recife, PE was 0.82% (95%CI: 0.68-0.97) [26]. In addition, this study found that of the 27 individuals aged under 25 who tested positive for HIV, 12 were classified as recent infections based on BED-CEIA. From 2007 to 2011, in a study of adolescents and young adults aged between 13 and 24 years old attending a CTA in Feira de Santana, BA was found an overall HIV prevalence of 1.94% in the population, specifically 3.0% among males and 1.6% among females [27].

#### Blood donors

Two articles studied the prevalence of HIV among blood donors. Between 1992 to 1999, the HIV prevalence in blood donors aged 18-25 in Curitiba, PR was 0.14% [28]. This age group presented the highest number of donations and the highest number of HIV cases (although HIV prevalence rates were higher amongst 26-35 year old donors). Between 2007 to 2013, after the implantation of NAT screening, HIV prevalence amongst blood donors aged 16-24 in Santa Catarina was 1.22% (95%CI: 1.01–1.46); an analysis of repeat donors in this age group showed an HIV incidence of 0.28 (95%CI: 0.20–0.37) per 1000 person-years [29]. The same article reported a sharp increase in HIV prevalence over time among 16-24 year old male donors; a similar rise was observed for young women until 2012, followed by a sharp decline.

#### Pregnant women

Sixteen studies reported HIV prevalence amongst pregnant women. In 1993, none of approximately 325 pregnant women aged 20 years or younger seen in an antenatal service in Recife were found to be HIV-positive [30]. One study measured HIV incidence amongst 106 women living with HIV seeking antenatal care in Sao Paulo, SP from 1991 to 2002 [31]. The authors estimated, based on STARHS, that 10% of those aged between 15 and 19, and 17.9% of those aged 20-24, years old had been infected within the past 6 months; however, this difference was not statistically significant. From 1996 to 1998, HIV prevalence for 10-20 year old pregnant females attending a teaching hospital in northern Parana was 1% [32]. In Campina Grande, PB in 2001, women testing for HIV during antenatal care, had an HIV prevalence of 0% amongst 14-19 year-olds and 0.07% amongst those aged 20-25 [33]. Between 2001 and 2002, postpartum women aged 15-20 in three public hospitals in Cuiabá, MS had an HIV prevalence of 0.5%, while those aged 21-25 had a prevalence of 0.4% [34].

In Mato Grosso do Sul state between 2002 and 2003, HIV prevalence among pregnant women aged 11-20 years old was 0.2% [35]. A prevalence of 0.5% (95%CI: 0.3–0.6) was seen in pregnant women aged 12-25 attending CTAs in southern Brazil in 2003 [36]. From 2003 to 2012, HIV prevalence amongst pregnant 11-18 year olds attending CTAs in Feira de Santana, BA was 0.31% [23]. In 2004-2005, a prevalence of 0.03% was found among pregnant women aged 12-19 who were seeking antenatal care in Goiania, GO [37]. Between 2004 and 2008 in Feira de Santana, BA, HIV prevalence among pregnant women testing for HIV during antenatal care aged <16, 17-19 and 20-24, was 0,3, 0,5 and 0,1% respectively [38]. Data from 2005 to 2013 found a HIV prevalence of 1.97% among pregnant teenagers aged between 10 and 19 with maternal anemia receiving prenatal care in Campinas, SP [39]. HIV prevalence in women during antenatal care in 2007 in Sergipe state was found to be 0.09% amongst women aged 10-19 (95%CI: 0.01-0.3) [40]. In Maceio, AL between 2007 and 2012, the HIV prevalence amongst pregnant women aged 15-26 years was 0.3% [41]. A nationwide analysis of 15-24 year-old women in labor in 2009 found a prevalence of 0.7% (95%CI: 0.4–1.1) [42].

In the northwestern region of Parana, in 2010, there was a prevalence of 0.3% among 14-19 year old women attending a teaching hospital [43]. A national study conducted in 2011-2012 found a HIV prevalence of 0.14% among pregnant women aged 12-19 [44].

#### Institutional settings

Four articles focused in youth in institutional settings. Between 1989 and 1991, HIV prevalence among 10-18 year olds admitted to a state-run shelter for homeless and youth offenders in Belo Horizonte, MG was 2% [45]. In 1994, a sample of 12-21 year old youths in a similar institution in São Paulo had an HIV prevalence of 10.3% for females and 2.6% for males [46]. In 2003, the prevalence of HIV infection amongst 19-24 years old in an institutional setting in Ribeirão Preto, SP was 0.09% [47]. In 2004-2005, only one case was found among 297 incarcerated youth in Salvador, BA, representing a prevalence of 0.34% (95%CI: 0.02–2.16) [48].

#### Men-who-have-sex-with-men

Six articles studied MSM. In a cohort of 18-50 year old MSM in Rio de Janeiro between 1995 and 1997, 18-19 year olds had an incidence rate of 8.4 (95%CI: 1.7-15) per 100 person-years, and 20-24 year olds had rate of 3.9 (95%CI: 1.7-6.1) per 100 person-years [49]. The same study found 18-24 year olds were significantly more likely to seroconvert than those aged 25-50 [adjusted Incidence Rate Ratio (aIRR) = 2.6 (95%CI: 1.3–5.6)]. In a stratified random sample of 30,970 literate 2002 Brazilian military conscripts, overall HIV prevalence was 0.09% (95%CI: 0.05-0.12%), while that amongst MSM was 0.56% (95%CI: 0.00-1.12%) [50]. In 2005 and 2006in the Campinas, SP metropolitan area HIV prevalence was 2.9% amongst 14-19 year-old MSM, and 5.9% in 20-24 year old MSM [51]. In a second sample of 2007 Brazilian military conscripts, HIV prevalence among MSM was 1.23% (95%CI: 0.34-2.13) compared to 0.11% (95%CI: 0.07-0.16) in the overall sample [52]. In 2010, prevalence amongst 18-24-year-old MSM in Belo Horizonte, MG was 2.8% [53]. Data from late 2011 and early 2012 reports a prevalence of 6.4% in MSM 18-24 year-olds in São Paulo, SP [54].

#### Female sex workers

Two studies considered female sex workers (FSW). In 2003 and 2004, there were 3 HIV-positive FSW in a sample of 44 FSW aged under 26 (6.8%) in Imbituba, SC [55], while a 2009 study of predominantly-female sex workers in the southern cities of Santa Catarina state, including Imbituba, reported a prevalence of 5.3% amongst the 57 individuals aged 18-24 [56].

#### Other groups

Male to female transsexuals aged 15 to 26 seeking sex reassignment surgery between 1998 and 2014 in from Porto Alegre, RS had an HIV prevalence of 14.8% [57]. In 1999, in Rio Branco, AC, a northern city in the Amazonian region with many indigenous citizens, no 12-21 year old attending an immunization campaign tested positive for HIV [58]. In 2002, none of the 70 women aged 18-21 attending a public family planning clinic in Salvador, BA were HIV seropositive; however 4% of 22-25 year-olds were HIV-positive [59]. In 2013, a study estimated the HIV incidence for 13-24 year olds in the general population in the cities of Recife, PE and Curitiba, PR to be 0.06% in both cities, using Sedia™ HIV-1LAg-Avidity tests [60].

### Risk exposures

Twelve studies provided information on risk exposures in age groups falling entirely within our inclusion criteria (i.e. 10–25 years old).

#### Counseling and testing center attendees

Four of the articles studying CTA attendees reported associations between risk factors and HIV infection. First, Bassols and colleagues reported in two articles that several sexual and drug behaviours were positively associated with HIV infection amongst CTA attendees in Porto Alegre [18, 19]. In the earlier study, HIV seropositivity was also positively associated with early sexual initiation (<12 years old) and unprotected sexual intercourse with a male partner (whether the respondent was male or female). Second, Bassichetto et al. showed that in Sao Paulo 78.9% of CTA users testing positive and aged 14-25 were single, and that sexual exposure was responsible for 98.7% of cases [21]. Although 40% of seropositive subjects were PWID, blood-to-blood transmission was not thought to be responsible for any infections in this sample. Third, Pereira et al. found the association between HIV infection and marital status to vary by gender for CTA attendees in Feira de Santana: 78.6% of HIV positive men were unmarried while 61.9% of infected women were married or in a stable relationship [27]. Among females, drug use, alcohol use, less than 8 years of schooling, reporting multiple partners and being married were associated with HIV infection. Among males, use of drugs other than alcohol, having more than 8 years of schooling and identifying as MSM were positively associated with HIV infection.

#### Pregnant women

One study in Campinas, SP found HIV-positive pregnant adolescents to have higher rates of anemia than their HIV-negative peers [39]. One national study found that among pregnant women in labor, living in the North region and an STI history were positively associated with HIV infection [42]. A third study found no significant association between age at pregnancy and HIV-seropositivity within 11-20 year olds in Mato Grosso do Sul state [35].

#### Institutional settings

Two studies studied associations between risk behaviors and HIV infection among adolescents in institutional settings. In Belo Horizonte, all three HIV-positive males in a study of street- and home-based youth between 1989 and 1991 reported heterosexual activity, and two of them reported injection drug use [45]. Amongst street-involved youth in São Paulo, HIV infection was associated with sex work for women, and history of STIs, Hepatitis C seropositivity and use of illegal drugs among men [46].

#### Other groups

Several predictors for HIV infection were reported among military conscripts in 2002: a positive syphilis test; identifying as MSM; any STI-related problem; more than 10 lifetime sexual partners; or residence in the South region [50]. By 2007, syphilis positivity and residence in the South region had ceased to be significant predictors, but the others remained [52]. Finally, 15-19 year olds had 2.4 times the odds of being HIV-infected compared to those aged 20-24 in a case-control study of HIV-positive women in Pelotas, RS [61].

## Discussion

In this study, we reviewed all published evidence on HIV prevalence and risk factors for infection amongst 10-25 year olds in Brazil. A key finding of our review is the lack of comprehensive data regarding risk behaviors for HIV infection either through studies specifically amongst adolescents, or stratified for this age group. Most of the studies we identified covered a broad age range, from adolescence up to senior ages, and in most cases HIV prevalence, but not risk factors, were stratified by age group. In some cases, the stratification grouped young and middle-aged adults in the same age group (e.g. 20 to 40 years old), which limited their usefulness for understanding risks amongst young people. While analyses of wider age ranges and the conditions of already-infected individuals appear quite common, focused evidence of the extent, and predictors, of young Brazilians’ risk of HIV infection remains limited. As a result, we were not able to conduct a quantitative assessment of any outcome using meta-analysis.

Despite its limitations, some important themes can be seen in the literature reviewed. First, the geographic coverage of the literature was limited. Of the 48 articles included in the final analysis, 14 were conducted in the South region and 14 in the Southeast region, while ten were in the Northeast, four in the Center-West and one in the North region of Brazil (four were national and one covered cities in both the South and Northeast). According to the last HIV Epidemiological Bulletin released by the Brazilian Ministry of Health in 2015, AIDS cases are increasing in the Center-West, North, and Northeast regions in the general population, and two of the four states with the highest AIDS reporting rates (Amazonas and Roraima) are in the North [62]. At the municipal level, the state capitals with the second to fifth highest reported AIDS rates (Florianópolis SC, Manaus AM, São Luís MA and Belém PA) have no published studies regarding young people and HIV. The literature’s focus in the South is supported by two of the three states of this region having the second- and third-highest reported AIDS rates in the country, and by the fact that almost 75% of the AIDS cases identified in Brazil from 1980 until June of 2015 were in the South and Southeast regions [7]. While these higher reporting rates may reflect these regions wealth, and therefore ability to diagnose AIDS cases, it may also reflect that the body of literature reflects past history of the HIV epidemic, rather than the current situation. It is also notable that many studies were conducted in state capital cities, and even those studies conducted elsewhere were often conducted in university campus cities, such as Campos dos Goytacazes [17], Feira de Santana [27, 38], Londrina [32] or Campina Grande [33]. All these trends highlight a clear need for additional research in non-traditional risk areas of Brazil.

Second, a large proportion of infections amongst both adolescents and young people were recent: 9.6% of HIV-positive individuals aged under 25 in Santos [15]; 17.9% of HIV-positive 20-24 year olds attending antenatal care in Sao Paulo [31]; 18.8% and 19.4% of HIV-positive 18-24 year olds Recife and Curitiba respectively in 2013 [60]; 25% of HIV-positive 15-24 year olds in Sao Paulo CTAs [21]; and 44.4% of HIV-positive adolescents seeking care in Recife CTAs [26]. While unsurprising, given the briefer sexual history of younger people and the predominantly sexual transmission route for HIV in Brazil, these data highlight the importance of developing preventive strategies focusing this age group, based on the behavioral risk factors to which they are more susceptible.

Third, the literature highlights some groups of young people at increased risk of HIV infection. HIV infection rates are high among MSM, and are rising relative to the general population. Two nationwide studies of military conscripts in 2002 and 2007 reported HIV prevalence among MSM of 0.56% and 1.23% respectively, while the overall population prevalence remained stable [50, 52]. HIV prevalence was even higher amongst sex workers, with an HIV prevalence of 6.8% [55] and 5.3% [56] amongst sex workers aged under 26 in two studies in Santa Catarina state. Sex workers are well-recognized as being at higher risk of HIV infection; HIV prevention policies focused on younger sex workers may be particularly important. Finally, there may be a downward trend in decreasing the infection rates amongst pregnant women, at least in one setting: HIV prevalence among pregnant women aged between 10 and 20 in Paraná in 1996-98 was 1% [32], but had fallen to 0.3% among 14-19 year old pregnant women by 2010 [43].

Fourth, several behaviors were reported as predictors of HIV infection. Use of illicit drugs or attending drug-using/selling places was consistently associated with increased HIV risk [18, 27, 46, 61]. In addition, a history of STIs was also associated with HIV infection in more than one study [42, 46, 50]. Interestingly, being in a stable relationship was associated with HIV infection among women while being unmarried was associated with infection among men [27]. This may reflect the higher risk for HIV amongst MSM. Somewhat surprisingly, in no study was past condom use associated with infection rates; this may reflect low interest amongst researchers, inaccurate reporting by respondents (due to recall bias or intentional mis-reporting) or truly no association in the populations studied.

One topic of concern within Brazil in recent years has been the “feminization” of the HIV epidemic. This was reflected in research highlighting that the male to female ratio of reported AIDS cases among adolescents in Rio de Janeiro city fell from 4.7 in the period 1984-1989 to 0.5 in 2005-2009 [63], and from 24 to two nationally between 1985 and 1999 [64]. HIV prevalence rates amongst 14-20 year olds were reported to have equalized by 2002 [65]. This led to the launch of a national campaign to combat the feminization of the epidemic in 2007, particularly through combatting women’s vulnerabilities [66]. More recently the ratio of male to female AIDS cases has again risen [7], however, young women living with HIV appear to be particularly vulnerable, even amongst all women living with HIV [67].

### Strengths and limitations

Our analysis has the strength of considering any study published on young people and HIV since the beginning of the epidemic. Furthermore, we considered studies in English, French or Portuguese. Nevertheless, there were some limitations to our work. First, we considered only published literature, and it is quite possible that other unpublished studies exist. We were also unable to access four of the 124 potentially relevant articles based on abstract review; however all of these were over 20 years old, and thus should not affect our review of recent epidemic trends. Second, our ability to make comparisons across time and space was limited by the highly varied study methodologies used; only when authors repeated their methods could we make direct comparisons. Furthermore, almost all studies grouped together wide age ranges, making it difficult to stratify our findings into the typically used five-year age categories (e.g. 15-19, 20-24). Generalizing beyond Brazil is also particularly difficult due to the unique history of HIV infection and care in this country. Finally, several of the studies used methods that are likely make it difficult even to generalize to the whole Brazilian population. For example CTAs are likely to have higher HIV prevalence since self-perceived risk for STI infection is a key predictor of attendance.

## Conclusion

Our review of the literature on HIV prevalence, incidence and risk factors in Brazil suggests that there is an unmet need for research into HIV risk patterns in the country. This is particularly true given suggestions in the literature that HIV prevalence may have been increasing among adolescents in recent years. The 2014 change in the law to require medical staff to notify the government of HIV infections may act as a spur to action for such research, by providing standardized nationwide data. However, given the relatively low national HIV prevalence, and thus relatively low HIV testing rates, amongst young Brazilians, the use of targeted, nationally representative surveys of young people using biological testing for HIV and STIs may well also be justified.

## Abbreviations

AC: Acre state
AIDS: Acquired immunodeficiency syndrome
aIRR: Adjusted incidence rate ratio
AL: Alagoas state
aOR: Adjusted odds ratio
BA: Bahia state
BED-CEIA: BED capture enzyme immunoassay
CTA: Counselling and testing center
FSW: Female sex worker
GO: Goiás state
HIV: Human immunodeficiency virus
MG: Minas Gerais state
MS: Mato Grosso do Sul state
MSM: Men-who-have-sex-with-men
NAT: Nucleic acid testing
OR: Odds ratio
PB: Paraíba state
PE: Pernambuco state
PR: Paraná state
PrR: Prevalence ratio
PWID: People who inject drugs
RJ: Rio de Janeiro
RS: Rio Grande do Sul state
SC: Santa Catarina state
SP: Sao Paolo state
STARHS: Serologic testing algorithm for recent HIV seroconversion
STI: Sexually transmitted infection
